# AI-powered VM selection: Amplifying cloud performance with dragonfly algorithm

**DOI:** 10.1016/j.heliyon.2024.e37912

**Published:** 2024-09-13

**Authors:** Sindhu Rashmi, Vikas Siwach, Harkesh Sehrawat, Gurbinder Singh Brar, Jimmy Singla, N.Z. Jhanjhi, Mehedi Masud, Mohammad Shorfuzzaman

**Affiliations:** a UIET, MDU Rohtak, India; b Lovely Professional University, Punjab, India; c School of Computer Science (SCS), Taylor's University, Subang Jaya, 47500, Malaysia; d Department of Computer Science, College of Computers and Information Technology, Taif University, P. O. Box 11099, Taif, 21944, Saudi Arabia

**Keywords:** Cloud computing, Cloud data center, Cloud service provider, Modified best fit decreasing, Virtual machine, Physical machine

## Abstract

The convenience and cost-effectiveness offered by cloud computing have attracted a large customer base. In a cloud environment, the inclusion of the concept of virtualization requires careful management of resource utilization and energy consumption. With a rapidly increasing consumer base of cloud data centers, it faces an overwhelming influx of Virtual Machine (VM) requests. In cloud computing technology, the mapping of these requests onto the actual cloud hardware is known as VM placement which is a significant area of research. The article presents the Dragonfly Algorithm integrated with Modified Best Fit Decreasing (DA-MBFD) is proposed to minimize the overall power consumption and the migration count. DA-MBFD uses MBFD for ranking VMs based on their resource requirement, then uses the Minimization of Migration (MM) algorithm for hotspot detection followed by DA to optimize the replacement of VMs from the overutilized hosts. DA-MBFD is compared with a few of the other existing techniques to show its efficiency. The comparative analysis of DA-MBFD against E-ABC, E-MBFD, and MBFD-MM shows %improvement reflecting a significant reduction in power consumption 8.21 %, 8.6 %, 6.77 %, violations in service level agreement from 9.25 %, 6.98 %–7.86 % and number of migrations 6.65 %, 8.92 %, 7.02 %, respectively.

## 1 Introduction

The old distributed computing scenario has entirely changed due to the quickly developing advanced technology known as cloud computing (CC). The deployment of virtualized IT resources using the internet where resources and services are being shared instead of having them on local servers would not be possible without cloud computing. Three categories used to group the services offered by the CC environment are Software as a Service (SaaS), Infrastructure as a Service (IaaS), and Platform as a Service (PaaS). In IaaS, Cloud Service Providers (CSPs) are the backend which consists of datacentres, servers, etc. CC is a paradigm known for its virtualization, which is responsible for the efficient load balancing and resource allocation and scheduling that enables the flexible and dynamic reservation and utilization of resources [1]. System virtualization technology forms the fundamental core of IaaS. It is explicitly crafted for expansion, providing extra computational capabilities to bolster data-heavy or computation-intensive applications. Simultaneously, these resources are provided as a service, allowing for resource sharing among multiple users. This aspect of resource sharing is a key factor in the profitability of cloud providers. However, the significant demand for cloud services can give rise to various resource management challenges that necessitate efficient handling of resources to cater to a multitude of users. Static allocation of resources would undoubtedly result in inefficient resource allocation, adversely impacting execution efficiency [2]. In the past, cloud service providers could efficiently assign preconfigured virtual machine (VM) instances to physical machines (PMs), enabling a single PM to host numerous VMs. However, the landscape has evolved. In addition to these preconfigured instances, there is now a user demand for the ability to incrementally scale specific resources, such as computing power, RAM, or storage, on a per-VM basis. The recent Gartner report published that the worldwide public cloud services end user spending has shown significant growth. Overall, it can be accessed that the total market growth accounts to 17.3 million US dollars in the year 2023 with is expected to reach 20.4 million US dollars and 22.1 million US dollars by the end of 2024 and 2025 respectively [3]. The earlier algorithms were primarily tailored for deploying fixed-resource VMs within the cloud and did not consider the dynamic provisioning of resources requested beyond the initial fixed VM configurations. Virtualization technology plays a pivotal role in energy conservation, making cloud servers designed with this technique highly attractive to cloud users [4]. During virtualization, effective management of VMs and PMs is crucial to ensure proper resource utilization which results in energy savings and it is done as, initially VMs are assigned to the PMs based on some balancing criteria which may be any of the existing ones. Then the capacity and load of each PM will be calculated based on a threshold value based on which PMs fall in the category of overutilized and underutilized PMs. If the PM is overutilized then based on some selection factor VM selection algorithm selects the VM from that PM and allocates it to the PM which has a better deciding factor. Thus, VMs are moved from overloaded PMs to underloaded ones. This paper explores the challenge of achieving optimal VM allocation that satisfies the needs of both cloud users and CSPs, offering insights into potential solutions [5].

### 1.1 Resource allocation

**Fig. 1:**
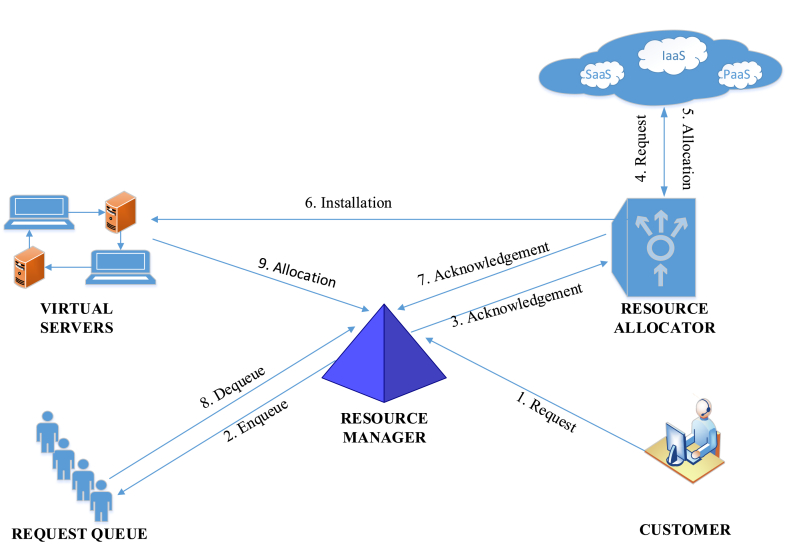
Resource allocation in CC.

When a user requests services from a cloud provider, the provider creates the necessary number of VMs and allocates resources to fulfill the user's request. Cloud providers offer services through static or dynamic provisioning. In static provisioning, the requested resources are pre-allocated, and the customer is billed on a monthly or annual basis. In contrast, dynamic provisioning provides resources on demand, and the customer is charged on a pay-per-use basis. A resource allocation process is shown in Fig. 1 below.

A resource allocation technique can be chosen by CSPs based on their different priorities such as [6].

1. Prioritizing customer's changing demands
2. Focusing requested resources
3. Optimization of the resources is the primary goal
4. For better performance task prioritization
5 . Efficient use of power

These techniques can be evaluated from both ends of CSPs and cloud service consumers (CSCs) based on a few parameters like utilization of resources, expenses, workload, energy consumption, service level agreement (SLA), and QoS from CSPs end, and waiting time, customer satisfaction, QoS, SLA and execution time from CSCs end. Utilization of resources: Optimizing resource utilization is crucial for cloud service providers to prevent resource idle time, promote environmental sustainability, and maximize profit. Expenses: Assessing the cost factor helps cloud service providers evaluate the profitability of different services, focusing on their profit and loss rather than that of the consumers. Workload: The burden on systems must be sufficient for the cloud service provider to guarantee that tasks are completed within the required time frame, which affects the scientific setting of utilization strategies. Waiting time: Task execution wait times are used to gauge system performance, thus both users and cloud service providers must keep wait times as low as possible. Execution time: Both users and cloud service providers benefit from minimizing execution time. However, multiple workloads on a single system may lead to interference and result in poor performance. Customer satisfaction: Effective resource allocation aims to maximize revenue and user satisfaction, which is a key goal for every cloud service provider. Energy Consumption: Minimizing energy consumption is essential for cloud service providers to support sustainable cloud services, considering the growing energy crisis. Cloud providers form VM groups within a resource pool, requiring the assignment of each VM to a host. This assignment poses a significant challenge in implementing IaaS. The constraints outlined in an SLA guide the association of VMs with individual clients.

However, resource allocation poses significant challenges in cloud computing. A handful of the challenges adjoining resource allocation are outlined below.

1. Inappropriate mapping of resources to VMs: Although virtualization is an effective technique, if resources are not properly mapped to VMs, it can negatively impact application performance. Using inadequate resource scheduling techniques can further exacerbate this issue.
2. Limited consideration of resource factors: Often, only a few resource factors are considered during the allocation process, which can be insufficient for effectively managing the cloud system.
3. Handling increasing request volumes: As the number of resource requests increases, it can lead to crises if the requests are not appropriately assigned to suitable VMs. Additionally, improper utilization of the CPU, either overutilization or underutilization, can further complicate the handling of requests.
4. Centralized resource manager: Many existing approaches assume a central resource manager to monitor and allocate VMs to PMs in a centralized manner, resulting in high system performance. However, this centralization introduces a single point of failure, compromising the system's robustness and making it vulnerable to disruptions [7].

These challenges highlight the need for efficient resource allocation strategies in cloud computing environments. Addressing these issues can enhance the overall performance and resource utilization in the cloud system. To tackle these challenges, the paper conducts a review of recent studies in the field that utilize Swarm Intelligence algorithms. Building upon this review, a novel framework is introduced to address the aforementioned issues. Furthermore, by offering ideas and methods to improve the energy efficiency of cloud-based apps, this work will be of significant assistance to future scholars in the field of cloud computing. Thus, the paper claims several contributions as follows.

• It employs DA to reduce false migrations by selecting suitable PMs for VM migration, thus resulting in effectively utilizing resources.
• It utilizes the Modified Best First Decreasing (MBFD) algorithm to prioritize VMs based on their resource requirements.
• Discusses the implementation of the Minimization of Migration (MM) algorithm to detect the overloaded and underloaded host.
• The DA approach is applied to optimize resource allocation to identify the best PM for migration.

These contributions show that the authors demonstrate an depth understanding of key ideas and techniques covered in the context of cloud computing resource optimization and performance improvement.

The rest of the article is structured as follows: Section 2 gives a thorough overview of the research that has been done in this area up to this point. The suggested layout is displayed in Section 3, which also explains the algorithm used. Section 4 compellingly presents the results of the proposed architecture, considering key parameters such as power consumption, SLA violation, migration count, and more. Finally, in section 5, a comprehensive summary is provided, underscoring the conclusion and the promising prospects of the proposed architecture.

## 2 Related work

Beloglazov et al., 2012 [8] significantly contributed to reducing data center energy costs and addressing environmental concerns in the Cloud computing industry. By introducing energy-aware resource allocation algorithms and demonstrating their effectiveness, it paved the way for enhancing the performance of cloud-based data centers. Honeyguide is an energy-efficient network design for lowering energy usage in data center networks with high redundancy requirements, according to IEEE Computer Society [9]. Through simulation-based research, Honeyguide proved that their approach to VM migration successfully lowered network power usage when compared to traditional VM migration strategies. OpenStack Neat's architecture and open-source implementation were presented by Beloglazov and Buyya, 2015 [10]. Within OpenStack clouds, OpenStack Neat functions as a dynamic VM consolidation framework that allows for the customization of VM consolidation methods. It seamlessly integrates with pre-existing OpenStack deployments, eliminating the need for configuration adjustments. Additionally, to promote research in dynamic VM consolidation, a benchmark suite was also proposed. This analysis package featured an evaluation approach for comparing and evaluating dynamic VM consolidation techniques, real-world workload traces, performance data, and OpenStack Neat as the primary software framework. VM Dynamic Forecast Migration (VM-DFM), a unique VM migration methodology that integrated dynamic prediction methods in a cloud environment, was proposed by Chen et al., 2015 [11]. By analyzing statistical data on VM memory consumption, VM-DFM forecasted the future memory requirements of VMs. This predictive information guided the decision-making process for selecting the most suitable VM to migrate, optimizing resource allocation, and improving overall system performance. Ali et al., 2016 [12] proposed a VM allocation algorithm called the Energy Efficient allocator (EE) algorithm that maximizes power efficiency by placing VMs on energy-efficient hosts. Abdelsamea et al., 2017 [13] a multiple regression algorithm, called Multiple Regression Host Overload Detection (MRHOD), had been developed to detect host overload. To detect overload, the method used CPU, memory, and bandwidth consumption as metrics. By considering multiple parameters instead of solely relying on CPU utilization, MRHOD provided a more accurate indication of host utilization. This method led to a notable decrease in energy consumption while consistently upholding SLAs at a high standard. Wu et al., 2017 [14] presented the THR_MUG scheduling technique, which combines a VM selection policy and a utilization threshold strategy. When an overloaded PM was identified, THR_MUG sought to identify eligible VMs for migration to keep the PM's utilization below the threshold. This strategy efficiently cuts down on energy use and the amount of VM migrations. During VM redeployment, Qiu et al., 2017 [15] took into account power consumption, communication delay, load balancing, and migration cost. The formalization of VM consolidation as a multi-objective optimization problem allowed for its solution with an enhanced genetic algorithm. Their strategy successfully trades off between optimized objectives and demonstrates higher overall performance compared to single objective optimization approaches, according to simulation experiments based on real-world workload traces. A VM consolidation method was presented by Mohiuddin and Almogren, 2019 [16]. The main focus of the method was reducing power usage by putting idle hardware servers into sleep mode. The research also addressed memory-related issues in edge data centers located in clouds that offer storage as a subscription service. The most important innovation was the VM migration mechanism, which was used in conjunction with a unique categorization system to ensure load distribution during allocation. The VM migration process aimed to consolidate VMs based on their workloads, reducing the count of physical servers, thereby mitigating energy consumption and promoting environmentally conscious computing practices. Dynamic Consolidation inspired by the Minimization of Migration Thrashing was developed by Liu et al., 2020 [17] to minimize migration thrashing and the overall amount of migrations. DCMMT achieves compliance with service-level agreements (SLAs) by strategically placing VMs susceptible to migration thrashing on the same physical servers instead of initiating migrations. Authors Yadav et al., 2020 [18] research proposal aimed to achieve two main outcomes in the cloud IaaS platform: 1) Minimizing electric energy consumption and reducing CO2 emissions to lower running costs and promote sustainable growth in the cloud computing industry, and 2) reducing SLA violations, reactivated hosts, and VM migrations through the introduction of novel least medial regression algorithms for overloaded host detection (LmsReg) and for VM selection minimum utilization prediction (MuP). These outcomes have shown significant potential in substantially minimizing energy consumption and optimizing resource allocation in data centers. Shalu and Singh, 2021) [19] presented an implementation of the E-MBFD Algorithm for VM allocation and an artificial neural network to cross-validate the allocation of VMs on PMs. The author effectively identified false allocations caused by inefficient resource utilization and facilitated the reallocation of these VMs. Jeevitha and Athisha, 2021 [20] introduced the shortest round vibrant queue (SRVQ) algorithm for VM scheduling, aimed to address waiting time and process starvation issues. The effectiveness of the SRVQ algorithm had been demonstrated, surpassing other existing algorithms. By improving resource management in cloud scheduling, the SRVQ algorithm enhances overall performance in the cloud computing paradigm. Kim et al., 2021 [21] proposed an approach named Min-Max Exclusive VM Placement (MMEVMP) which focused on the cloud environment for scientific data, where data-intensive jobs requiring disk operations are common. They claimed to minimize both service level agreement violation (SLAV) and energy consumption [22]. To increase the efficiency of current server hardware and workloads by transferring them to virtual server hardware, a server consolidation technique was implemented by Uddin et al., 2021 [22]. This technique made it possible to create green and energy-efficient cloud data centers. To effectively manage and distribute workloads from physical server machines to virtual server machines, a novel Virtualized Task Scheduling Algorithm was also introduced. The effectiveness of the proposed technique and algorithm was evaluated through a case study conducted in Pakistan, specifically on a tier-level data center. Using VM consolidation, Arshad et al., 2022 [23] proposed the Energy Efficiency Heuristic algorithm. The goal of this study was to reduce power usage in a cloud-based system without sacrificing service quality. To reduce energy consumption, the author divided hosts into three groups depending on thresholds and redistributed VMs. Evaluation of the algorithm's performance took into account execution time, service level agreement violations, performance deterioration, and VM migrations. Tran et al., 2022 [24] presented a conceptualization of the virtual migration problem employing game theory principles, aiming to attain load balancing and optimize resource utilization. An algorithm named V2PQL was introduced for VM migration. This algorithm harnesses the power of the Markov decision process and the Q-learning algorithm. An innovative VM migration algorithm, named Preserving “Resource Handiness and Exigency-Based Migration” (PRH-EM), was introduced by Karthikeyan et al., 2023 [25] to strike a balance between lowering energy consumption and maximizing profit. In the initial phase, the authors classified data centers based on MIPS and cost value, factors that significantly influence resource availability. They made a comparison between data centers' total workload capability and each VM's capacity, allowing for quick migrations during spikes in demand to avoid wasted resources. The migration strategy, built around managing overutilization and underutilization, effectively reduced resource usage, leading to lower energy consumption and carbon emissions. Additionally, the proposed approach ensured uninterrupted resource availability and service delivery to users, even in the event of VM failures, ultimately increasing profitability. Kaur et al., 2023 [26] had reviewed the role of cloud computing in the IT sector, highlighting global deployments by tech giants like Google, Microsoft, and Facebook. Reducing power consumption was initially overlooked due to performance demands on cloud services (IaaS, PaaS, SaaS). Researchers had later introduced power-efficient algorithms, including statistical learning and bioinspired methods. The paper had focused on improving virtual machine migration approaches and proposed an energy-efficient VM allocation and migration algorithm inspired by the Smart Elastic Scheduling Algorithm (SESA). By incorporating cosine similarity and bandwidth utilization, the proposed method had aimed to enhance QoS. The evaluation had compared overall power consumption and SLA violations against existing techniques, presenting a new algorithm to address identified issues. Buyya et al., 2024 [27] had reviewed the critical role of cloud computing as essential infrastructure for the modern economy, providing subscription-based services on a pay-as-you-go basis. The exponential growth of cloud computing had led to significant energy consumption, with data centers projected to use 20 % of global electricity and emit 5.5 % of carbon emissions by 2025. This energy use also caused operational issues, including reduced system reliability and increased cooling needs. To address these challenges, the paper had proposed a learning-centric approach for integrated management of next-generation cloud environments, aiming to reduce energy consumption and carbon footprint while maintaining service quality. The conceptual architecture and early results had demonstrated potential benefits for enhancing energy efficiency and sustainability. Yenugula et al., 2024 [28] concluded that fostering sustainable cloud computing had been crucial for a sustainable future. Cloud computing's environmental impact could not have been overlooked. Implementing energy-efficient designs, using renewable energy, optimizing cooling systems, and educating stakeholders had been essential strategies. These practices had reduced energy consumption, lowered costs, improved efficiency, and enhanced reputation, helping organizations meet sustainability goals and fulfil social responsibilities. Individuals also had a role in adopting energy-efficient behaviours and choosing sustainable providers. Achieving sustainable cloud computing had required collaboration among all stakeholders, ensuring cloud computing could continue to support the digital economy sustainably.

### 2.1 Optimization of resource allocation

**Fig. 2:**
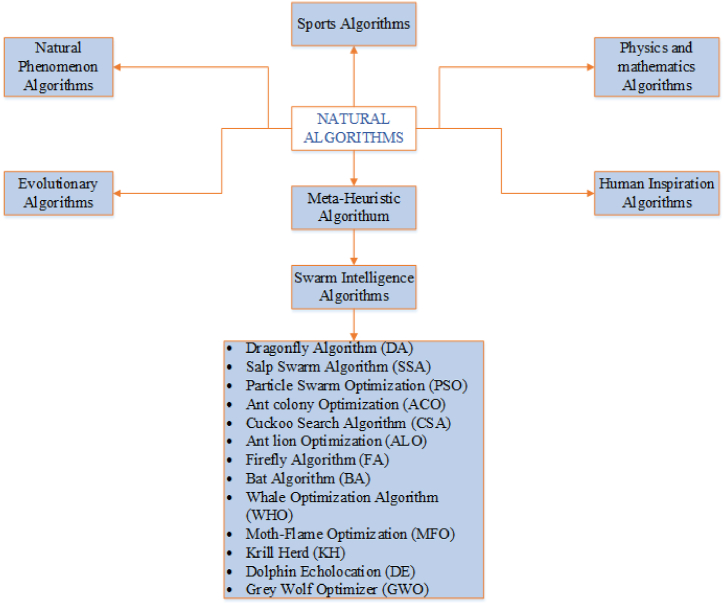
Classification of natural computing.

The optimization process can be applied to various systems, including IoT devices and WSNs, with the aim of either minimizing or maximizing their current state. Optimization can be achieved through population manipulation, which involves rejecting, selecting, or replacing populations. The population refers to a group that is selected for the optimization process. The selection and rejection of populations are based on specific criteria that determine which populations are chosen or discarded. However, when it comes to population replacement, a decision is made regarding which population should be replaced and with what new population. It is worth noting that the replacement process can potentially increase the execution time of the optimization process. The concept of optimization is inspired by nature itself and is often referred to as natural computation. It can be categorized into six main categories, as illustrated in Fig. 2.

In this article, our primary focus will be on the third category, which pertains to meta-heuristic algorithms, particularly swarm intelligence (SI) optimization algorithms. The remarkable collective social behavior exhibited by physical/chemical systems, organism swarms, or communities such as herds of animals, flocks of birds, or colonies of insects serves as a profound inspiration for the development of SI algorithms. Any natural behavior that relies on group dynamics for the successful execution of a process falls within the realm of SI. Notably, insects like honeybees, fireflies, dragonflies, ants, and mosquitoes display this group behavior, driven by their innate goal of attaining high-quality food despite their limited cognitive abilities. Leveraging this inspiration, we can design algorithmic architectures that are well-suited for various machine-learning processes. Several SI algorithms which have been used by the research in the field of resource allocation and balancing of load on the resources in recent years are analyzed to present and summarized in Table 1 below.

**Table 1:** Comparative analysis of various Optimization algorithms used for resource allocation in data centers.

Paper reference	Authors	Year	Algorithm used	Findings of the article	Research gaps
[29]	Muthulakshmi B et al.	2019	Artificial Bee Colony (ABC), Simulated Annealing (SA)	Improved scheduling and resource allocation in cloud environments	Lack of comparative evaluation with existing scheduling and resource allocation algorithms. Limited analysis of the algorithm's performance under different workload conditions.
[30]	Rb et al.	2021	Particle Swarm Optimization (PSO)	Improved VM selection in cloud computing environment	Insufficient comparison with other VM selection techniques. Limited investigation of the algorithm's scalability and robustness.
[31]	Shafiq et al.	2021	Load Balancing algorithm	Optimized load balancing for cloud computing applications in data centers.	Lack of detailed analysis of the algorithm's performance, scalability, and applicability to different types of workloads.
[32]	Ullah et al.	2019	ABC	A comprehensive review of the applications of the ABC algorithm for load balancing in cloud computing.	Limited examination of the algorithm's limitations and potential improvements.
[33]	Kansal et al.	2015	ABC	Improved energy-aware resource utilization in cloud computing environments using the Artificial Bee Colony algorithm.	Lack of in-depth analysis of the identified gaps and limitations in the research paper.
[34]	Parthiban et al.	2022	Chaotic Salp Swarm Optimization	Energy-aware VM placement technique using Chaotic Salp Swarm Optimization for cloud data centers.	Insufficient investigation of the algorithm's performance under different load conditions and its scalability.
[35]	Meraihi et al.	2020	DA	A comprehensive review of the Dragonfly algorithm and its applications.	Lack of critical analysis of the algorithm's limitations, potential challenges, and future directions.
[36]	Polepally et al.	2019	Dragonfly optimization	Optimized load balancing in cloud computing using Dragonfly optimization and constraint measure-based approach.	Limited discussion on the algorithm's performance in large-scale cloud environments and its adaptability to different workloads.
[5]	Talwani et al.	2021	Bee Colony algorithm (BCA)	Improving Energy Efficiency in VM Migration within Cloud Computing Environments through an Enhanced Bee Colony Approach.	Lack of comparative evaluation with other energy-efficient VM migration techniques.
[37]	Devaraj et al.	2020	Firefly algorithm, Multi-Objective PSO	To achieve energy-efficient load balancing, combine enhanced multi-objective particle swarm optimization with firefly hybridization.	Insufficient investigation of the algorithm's performance in dynamic and heterogeneous cloud environments.
[38]	Selvaraja et al.	2019	Analogous Particle Swarm Optimization (APSO)	Enhancing VM Selection for Anomaly Detection in Cloud Environments through Swarm Intelligence.	Lack of specific algorithms and gaps mentioned in the provided information.
[39]	Madhumala et al.	2021	Modified First Fit Decreasing Algorithm (MFFD) with PSO	The Fusion of Modified First Fit Decreasing Algorithm (MFFD) and Particle Swarm Optimization (PSO) Algorithm for Optimal VM Placement on Active PMs, with the Aim of Minimizing Energy Consumption.	Lack of comprehensive comparative analysis, limited generalizability, and absence of real-world deployment validation.
[40]	Kassanuk T et al.	2023	Bird Swarm Optimization (BSO) and DA	By combining dynamic degree balance, CPU-based VM allocation, and hybrid BSO-DA, the proposed algorithm optimizes system performance while preserving balance.	Lack of energy efficiency factor which is major in today's scenario also live VM migration factor is excluded from the study
[41]	Tripathi A et al.	2020	Modified DA	A customized algorithm of Dragonfly is employed to enhance resource utilization by optimizing the placement of VMs within cloud data centers.	In the dynamic environment of cloud computing, the author fails to address the challenge of continuously generating new VM requests and terminating older ones.
[42]	Amini Z et al.	2018	Dragonfly Optimization	The resource allocation and system performance in CC are improved by the load balancing solution that is suggested and is based on the dragonfly optimization algorithm.	lack of comparative evaluation, Limited scalability analysis, inadequate assessment of robustness and adaptability.
[43] [44]	Latchoumi et al. Gong et al.	2022 2024	Quasi-Oppositional Dragonfly Algorithm for Load Balancing (QODA-LB) LSTM + GRU using Deep reinforcement Learning	Developed a novel Quasi-Oppositional Dragonfly Algorithm for Load Balancing (QODA-LB) intending to achieve optimal resource scheduling within a CC configuration. Furthermore, this algorithm improves the convergence rates of the Dragonfly Algorithm (DA). Machine learning models improve resource allocation and performance in cloud environments.	N/A Implementing and integrating these models can be computationally intensive and technically challenging.

### 2.2 Problem statement

We aim to address the challenge of resource optimization in cloud computing environments by formulating a sophisticated problem statement for the selection of VMs from a pool of potentially overutilized PMs. Our objective is to intelligently allocate VMs to underutilized PMs, thereby reducing resource congestion and enhancing overall system performance. To accomplish this, we define two mathematical equations. The first equation calculates the resource utilization ratio ($U_{\text{pm}}$) of a PM, which represents the ratio of the current resource usage ($R_{\text{used}}$) to the maximum capacity (R-max) of the PM and then introduces the VM placement score ($S_{\text{vm}}-\text{pm})$ as an assessment of the suitability of placing a VM on a PM. This score depends on the PM's utilization ratio ($U_{\text{pm}}$) which is referred to by the CPU utilization, and, the resource requirements ($R_{\text{req}}$) of the VM. It is formulated using weighting factors (α and β) to determine the relative importance of the PM's utilization ratio and the VM's resource requirements. The second equation formulates the optimization objective, which aims to maximize the overall system performance while minimizing resource congestion. This objective is achieved by maximizing the sum of the placement scores ($S_{\text{vm}}-\text{pm}$) for all VM-PM pairs. By integrating these mathematical equations into our problem statement, we provide a detailed and sophisticated framework for optimizing resource allocation in CC, promoting efficient VM selection, and improving system performance.

The problem addressed in this research work is dependent upon VM placement score ($S_{\text{vm}}$-pm) to evaluate the suitability of placing a VM on a suitable PM. This score is determined by considering both the PM's utilization ratio ($U_{\text{pm}}$) and the resource requirements of the VM ($R_{\text{req}}$). We incorporate weighting factors (α and β) to reflect the relative importance of these factors in the scoring process. The criteria for selecting the weights α and β in equation (1) depend on the relative importance of the PM's utilization ratio $U_{\text{pm}}$ and the VM's resource requirements $R_{\text{req}}$ in determining the VM placement score $S_{\text{vm}}-\text{pm}$ using reference [26]. The equation for calculating the placement score is given by eq. (1):

(1) $$S_{\text{vm}}-\text{pm}=\alpha \;*\;U_{\text{pm}}+\beta \;*\;\left(1\;-\;\frac{R_{\text{req}}}{R-\max\;}\;\right)$$

In this equation, the term α ∗ $U_{pm}$ encourages the selection of underutilized PMs by giving higher scores to PMs with lower utilization ratios. The term β ∗ $\left(1\;-\;R_{req}/R-\max\;\right)$ incentivizes the selection of PMs that have sufficient available resources to accommodate the VM, as a lower value of $R_{req}\;/\;R-\max$ indicates a higher availability of resources.

To define the optimization objective, we aim to maximize the overall system performance while minimizing resource congestion. This can be achieved by maximizing the sum of the placement scores for all VM-PM pairs. Mathematically, the optimization objective is formulated as eq. (2):

(2) $$\text{Maximize}\sum \left(S_{\text{vm}}-\text{pm}\right)$$

Here, the summation is taken over all VM-PM pairs, and the objective is to maximize the sum of their placement scores, thereby encouraging the selection of VM-PM pairs that contribute to optimal system performance and resource utilization.

The solution to our problem presents a detailed and sophisticated framework for addressing the selection of VMs from overutilized PMs. This formulation enables the intelligent allocation of VMs to underutilized PMs, leading to improved resource efficiency and overall system performance in cloud computing environments.

## 3 Proposed work

**Fig. 3:**
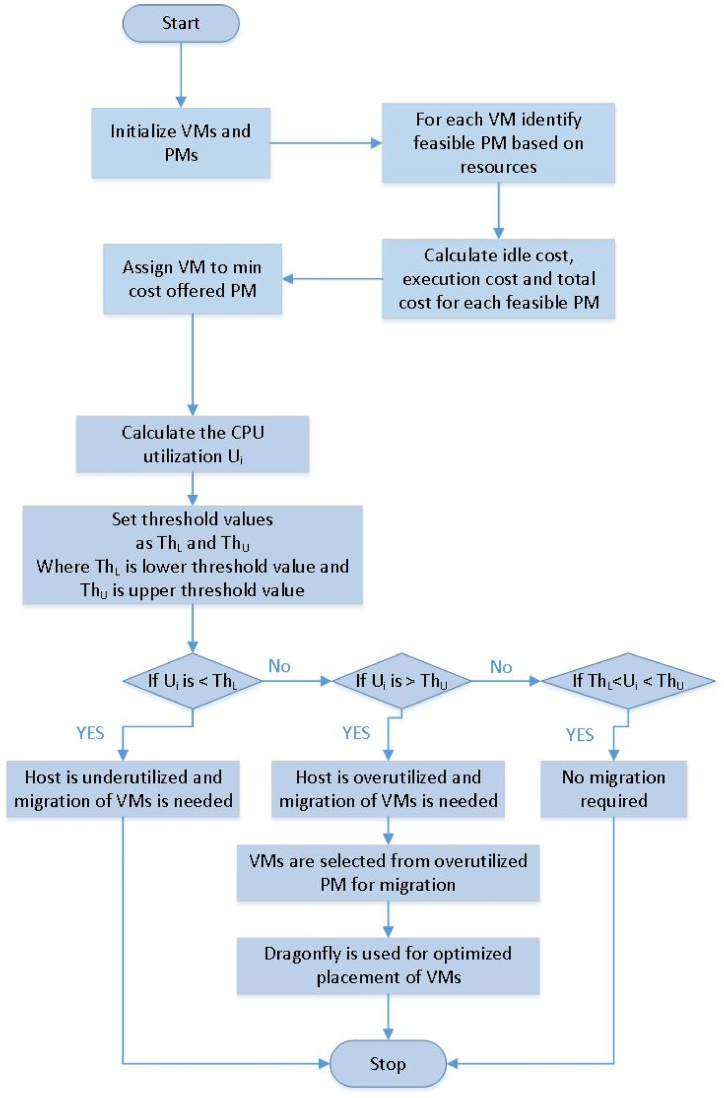
Flowchart of DA-MBFD

This research aims to optimize data center profitability through enhanced energy efficiency. This objective is realized by minimizing the overall energy consumption of both idle and operational nodes, which host VM requests from users. The challenge of VM consolidation can be classified into three distinct types: (i) VM placement (ii) identification of overloaded and underloaded hosts, and (iii) selecting the VMs from the selected overloaded PM. VM placement can further be distinguished into two: first is the accommodation to a newly requested VM over the PM, and second is the optimization of the allocated VM (Beloglazov et al., 2012). This article focuses on the optimization of the allocated VM using the dragonfly algorithm and the output of their performance parameters are computed. The framework of the proposed work named DA-MBFD is shown in Fig. 3 below.

### 3.1 VM placement over PM

Consider a Cloud Data Center (CDC) consisting of ‘P’ number of PMs (P_1_, P_2_, P_3_, P_4,_ … …, P_p_) using N number of VMs as (V_1_, V_2_, V_3_, V_4_, … … ….V_N_). Every VM (V_i_ where i = 1,2, … … N) has some CPU utilization (U_i_), memory, and clock frequency (C_f_) also it consumes some power (P_i_). A cloud system's processing capacity is determined by the amount of instructions processed per second, which is directly correlated with the CPU clock frequency (C_f_). Suppose, M number of users are submitting their request to the CDC as (U_1_, U_2_, U_3_, … … ….U_M_). The system rejects requests for VMs if the requested resources are not available after the broker in the CDC receives the VM requests sent from different users to identify the available PMs based on the users' requirements. The equation contains the accepted VM request from the CDC eq. (3) as

(3) $$R_{A}\;\text{of}\;‘P’\;\text{PMs}=\text{OR}\;‐\;\int_{i=1}^{\text{TA}}\;{\mathrm{PR}}_{P}$$

here R_A_ is the available resources, OR is the original number of resources, TA is the total number of allocations to the pth PM, and PR_P_ is the only remaining resources of the pth PM.

Also, a “Request Completion Ratio” [5] (RCR) is also computed according to eq. (4). RCR is used by the CDC to rank its PMs to serve the users with VM requests.

(4) $$\text{RCR}\;\left({\text{VM}}_{R}\right)=\frac{\text{RC}}{\text{AR}}$$

where RC denotes the demands that the PM fulfilled without violating the SLA AR denotes the total number of requests that were allotted and VM_R_ is the number of VM requests submitted.

Once user requests are collected, the core responsibility of the Cloud Data Center (CDC) is to allocate VMs to PMs using VM deployment techniques. In this research, we adopt the MBFD approach for VM deployment. This method entails arranging VMs in descending order of CPU capacities and assigning them to the PM with the least power consumption. By prioritizing the selection of the most power-efficient node, this algorithm strives to optimize energy efficiency during the VM allocation process. MBFD algorithm is given below.

**Figure:**
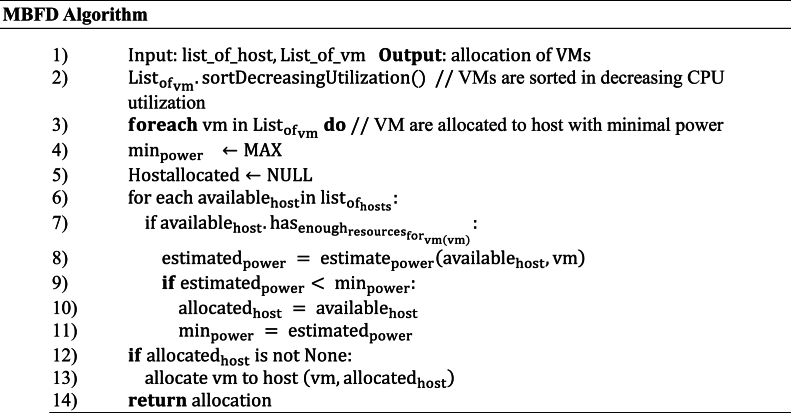

### 3.2 Calculating over and underutilized PM

Power model ($P_{s}$) for a server is mathematically represented by eq. (5)

(5) $$P_{s}=\text{static}+\text{CPU}\;\text{utilization}\times \text{dyanmic}$$

Where ‘static’ is the power consumed by the server in the idle state, an idle server consumes 70 % of the maximum power [5], illustrated in eq. (6) where ‘f’ is the fraction of idle server power consumption i.e. (70 %). ‘dynamic’ is the power consumed by the server in its active state illustrated in eq. (7). The elaborated power model is in eq. (8),

(6) $$P_{\text{static}}=f.P_{\max}$$

(7) $$P_{\text{dynamic}}=\left(1-f\right).P_{\max}$$

(8) $$P_{s}=P_{\text{static}}+U_{i}\times \left(P_{\max}-P_{\text{idle}}\right)$$

Therefore total power $P_{total}$ consumed by a server in the time interval of (0,t) is represented in eq. (9).

(9) $$P_{\text{total}}=\int_{0}^{t}P_{s}\;t.\text{dt}$$

### 3.3 Optimized replacement of VM is done using the DA algorithm

**Fig. 4:**
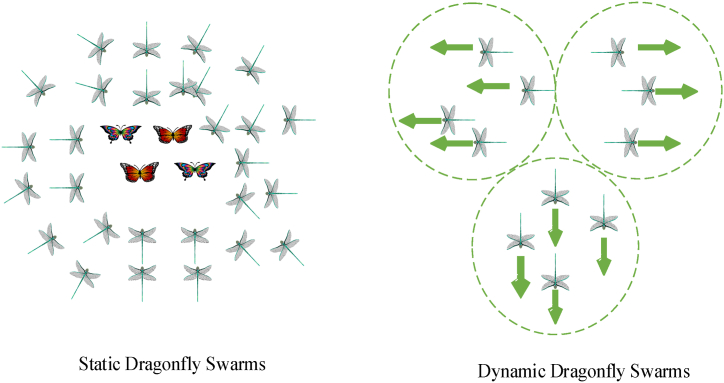
Illustration of static and dynamic swarms.

One of the most recent metaheuristic SI algorithms, Dragonfly is used to tackle a range of optimization issues. It takes its cues from the static and dynamic behavior of dragonflies in the wild. During the exploration phase, the dragonflies in the static behavior form a static swarm. They congregate in tiny groups and hunt for prey by circling a small area back and forth. The dragonflies move into the exploitation stage of their dynamic behavior. They gather into a sizable swarm and go far in a single direction, away from adversaries. Fig. 4 below depicts the dragonflies' static and dynamic behaviors.

The miniature swarm behavior of the dragonflies is illustrated with the help of five primordial principals, these are Alignment (A), Separation (S), Cohesion (C), Attraction to food (F), and Distraction from the enemy (E).

(A) indicates the analogy between the individual's velocity and the neighboring individual's velocity of the same group. (A) is indicated by eq. (10) where M is the number of individuals [35].

(10) $$A_{i}=\frac{\sum_{j-1}^{M}V_{j}}{M}$$

(S) is the stagnant collision that needs to be followed by individuals to evade collision with other individuals in the neighborhood. (S) is indicated in eq. (11) where P is the position of the current individual and P_j_ is position of the jth neighboring individual [35].

(11) $$S_{i=-}\sum_{j-1}^{M}P-P_{j}$$

(C) is the probability with which an individual is engaged with the center of the swarm's group. (C) is indicated in eq. (12).

(12) $$C_{i=}\frac{\sum_{j-1}^{M}P_{j}}{M}-P$$

(F) is the difference between the current individual position and the food source position which is indicated by eq. (13). (E) is indicated in eq. (14).

(13) $$F_{i=}F_{p}-P$$

(14) $$E_{i}=E_{p}+P$$

**Fig. 5:**
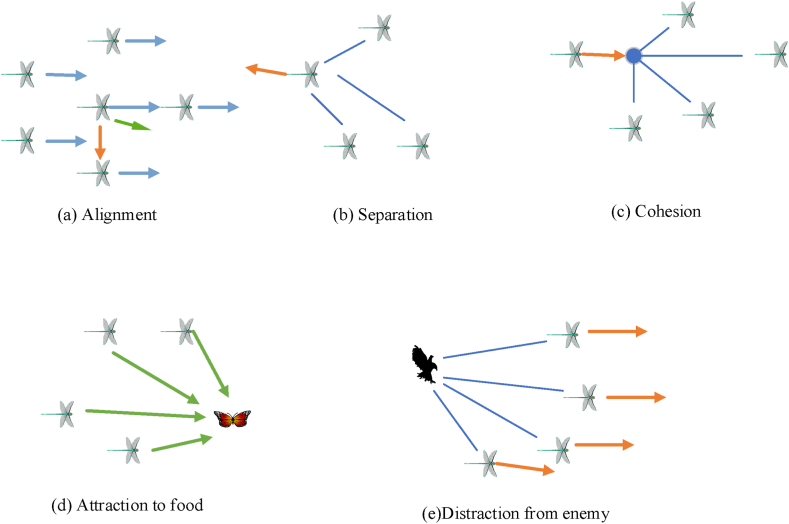
Principals of swarm behavior of Dragonflies (a) alignment, (b) separation (c) cohesion (d) attraction (e) distraction from the enemy.

These principles are illustrated in Fig. 5 below. The principals of the swarm intelligence behaviour of dragonflies is further illustrated using Fig. 5. Fig. 5(a) presents the alignment of files, 5(b) presents separation, 5(c) presents cohesion, 5(d) attraction towards food while 5(e) presents distraction from the enemy. Based on the velocity/step vector ΔP the dragonflies inside their search space are given by orientation vector P and updated as in eq. (15).

(15) $$\Delta P_{i}^{t+1}=\left(sS_{i}+aA_{i}+cC_{i}+fF_{i}+eE_{i}\right)+w\Delta P_{i}^{t}$$

Where ‘s' stands for the ${\text{separation}}_{\text{weight}}\text{,}$ ‘Si’ for the individual's separation, ‘a' for the $\text{alignmen}t_{\text{weight}}$, ‘Ai’ for the individual's alignment, and ‘c' for the ${\text{cohesion}}_{\text{weight}}$ ‘F' stands for the ${\text{food}}_{\text{factor}}$, and ‘Ci’ stands for the coherence of the individual i. ‘Fi’ denotes the individual's food source, ‘e' denotes the enemy component, and ‘Ei’ marks the enemy's position. ‘w' inertia weight, while ‘t' stands for the repetition number. As a result, the dragonfly's position at time point plus one is updated according to eq. (16).

(16) $$P_{i}^{t+1}=P_{i}^{t}+\Delta P_{i}^{t+1}$$

High alignment and low cohesion weights are incorporated to aid the exploring phase, as suggested by Meraihi et al., 2020 [35]. In contrast, using moderate alignment and elevated cohesion weights ensures the exploitation phase. The weights s, a, c, f, e, and w of the Dragonfly Algorithm (DA) are capable of being dynamically tuned to control how quickly the algorithm converges. When there are no surrounding solutions accessible, a random walk, especially a Levy flight, is implemented to improve exploration, add randomization, and make exploitation easier within the dragonfly algorithm. As a result, the dragonfly's position at iteration t+1 is updated as shown in eq. (17).

(17) $$P_{i}^{t}=P_{i}^{t}+\text{levy}\;\left(d\right)\times P_{i}^{t}$$

**Fig. 6:**
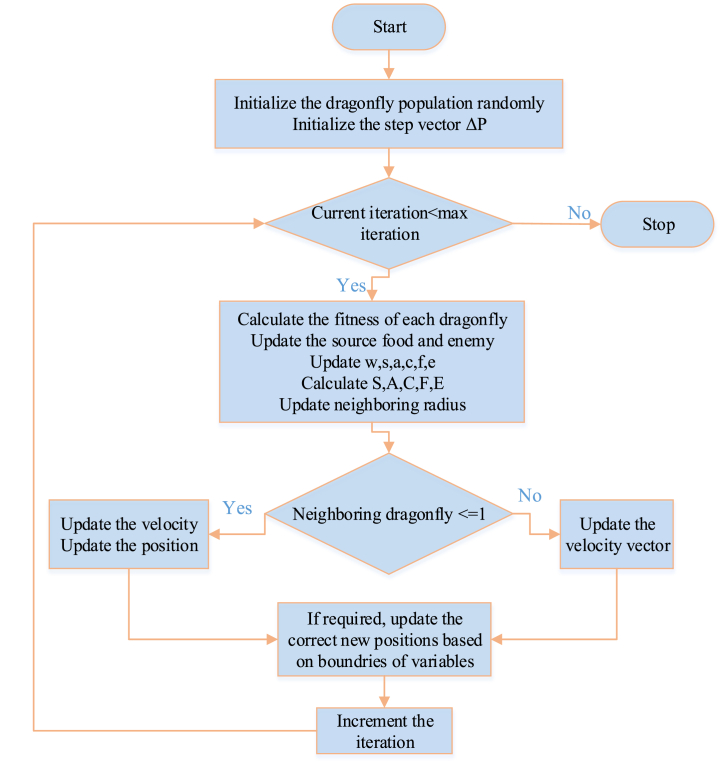
Flowchart for dragonfly algorithm.

The operation of the DA is depicted in the flowchart shown in Fig. 6 below.

**Figure:**
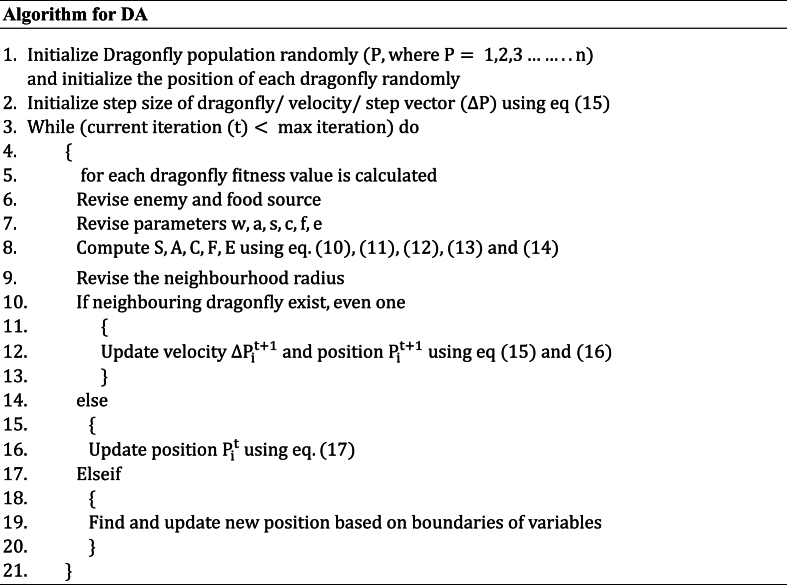

The proposed work is carried out on a simulation setup with a variable number of PMs and VMs in a CDC. The simulation results are presented in section 4 below.

## 4 Results and discussion

This section details the effectiveness of the proposed work, DA-MBFD, and compares it to a select handful of the existing algorithms, including E-ABC [11], MBFD-MM [20], and E-MBFD [21]. The proposed work is carried out on Java with the CloudSim toolkit and requires the PC with the configuration of 4 GB RAM, Windows 10 OS, and the Intel processor-number of cores 7 ((Core™) i7 CPU) - Q 720@1.60GHz, where we have taken [20–500] PMs and [100–2500] VMs. The performance of the proposed work is based on the power consumed in KW-h, SLA-violation, and the migration count. To execute the proposed work, a few assumptions are listed in Table 2. The obtained results of the proposed work and its comparison with the existing algorithms are listed in Table 3. The simulation is repeated 10,000 times considering up to 2500 VMs.

**Table 2:** Required assumptions of parameters.

Number of PMs	500
Number of VMs	2500
RAM	8000
CPU Utilization	3ghz
Number of CDC	1
PM storage (MIPS)	100000

**Table 3:** Comparison of computed parameters.

No. of PMs	No. of VMs	Power consumption in KW-h				SLA-Violation				Migration Count			
		DA-MBFD	E-ABC	E-MBFD	MBFD-MM	DA-MBFD	E-ABC	E-MBFD	MBFD-MM	DA-MBFD	E-ABC	E-MBFD	MBFD-MM
20	100	8.44	9.82	8.63	9.83	0.08	0.09	0.09	0.09	35	39	36	39
40	200	8.90	9.67	10.18	9.12	0.08	0.10	0.10	0.10	63	63	71	70
60	300	9.30	10.57	10.55	9.96	0.08	0.09	0.10	0.09	83	83	84	91
80	400	9.74	10.29	11.40	9.80	0.08	0.09	0.10	0.10	107	113	114	115
100	500	10.18	11.56	10.68	11.42	0.09	0.09	0.09	0.09	173	180	188	178
120	600	10.63	11.11	11.59	11.96	0.09	0.10	0.10	0.09	159	164	179	164
140	700	11.08	11.57	12.94	11.78	0.08	0.09	0.09	0.09	200	220	218	213
160	800	11.57	12.16	13.14	13.14	0.09	0.09	0.09	0.09	242	265	276	246
180	900	11.86	12.86	11.88	12.58	0.08	0.10	0.09	0.09	240	257	249	259
200	1000	12.34	14.01	13.35	12.53	0.09	0.10	0.09	0.10	304	340	308	320
220	1100	12.46	14.62	12.47	14.20	0.09	0.10	0.09	0.09	304	333	324	337
240	1200	12.91	13.52	13.25	13.23	0.09	0.10	0.10	0.09	402	432	423	427
260	1300	13.46	13.65	14.04	13.76	0.09	0.09	0.09	0.09	442	492	475	468
280	1400	13.84	16.08	14.76	14.11	0.09	0.10	0.09	0.10	364	370	415	406
300	1500	14.43	14.59	15.04	15.35	0.09	0.09	0.09	0.09	472	513	518	498
320	1600	14.84	15.63	16.83	15.34	0.09	0.09	0.10	0.10	525	530	598	574
340	1700	15.21	16.41	16.44	16.05	0.09	0.10	0.10	0.10	535	559	600	569
360	1800	15.52	18.14	17.96	17.05	0.09	0.10	0.09	0.09	535	550	603	548
380	1900	15.81	16.30	16.46	17.05	0.09	0.10	0.09	0.09	647	723	690	656
400	2000	16.26	17.45	18.00	17.03	0.09	0.10	0.09	0.09	559	580	595	593
420	2100	16.49	17.27	18.87	16.58	0.09	0.11	0.10	0.10	575	607	658	613
440	2200	17.37	18.10	18.90	19.32	0.09	0.10	0.10	0.11	558	617	634	611
460	2300	17.31	18.13	18.36	19.43	0.09	0.10	0.09	0.10	689	753	763	764
480	2400	18.67	19.79	21.02	19.33	0.08	0.10	0.09	0.09	817	877	893	901
500	2500	19.01	20.98	19.63	19.83	0.10	0.10	0.10	0.10	870	944	935	919

### 4.1 Comparative analysis

**Fig. 7:**
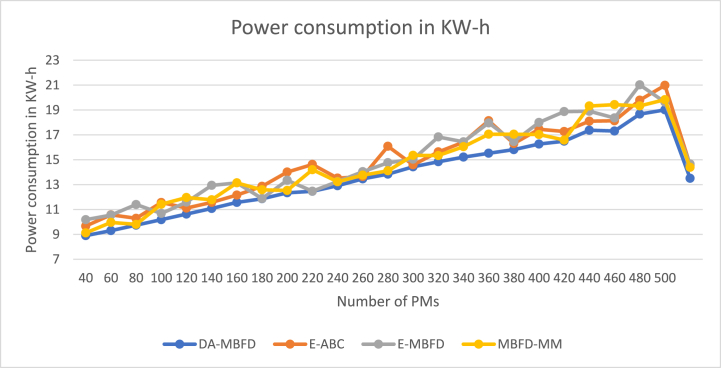
Power consumption.

We have compared the performance of the DA-MBFD algorithm against three major parameters power consumed, SLA Violation, and the number of VM migrations. The rise in power consumption concerning the increase in the number of PMs is given in Fig. 7. The figure also shows that power consumed by the proposed DA-MBFD is least among the other studies used in the analysis. This is majorly with the reduced wasteful VM migrations.

This section also illustrates the % improvement of DA-MBFD over E-ABC. E-MBFD and MBFD-MM corresponding to these parameters. The analyzed improvement is presented in the figure below.

**Fig. 8:**
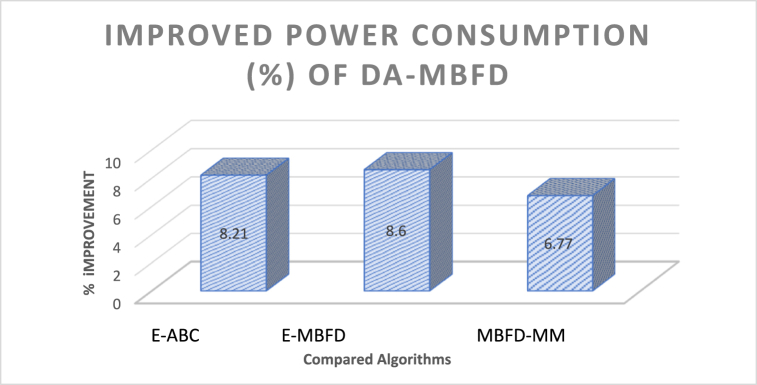
Improvement in power consumption.

Fig. 8 illustrates the % Improvement in power consumption of DA-MBFD against E-ABC, MBFD-MM, and E-MBFD concerning VMs as DA exhibits superior optimization data exploration capabilities. Energy saved using DA-MBFD is 8.21 %, 8.6 %, and 6.77 % compared to E-ABC, E-MBFD, and MBFD-MM respectively.

**Fig. 9:**
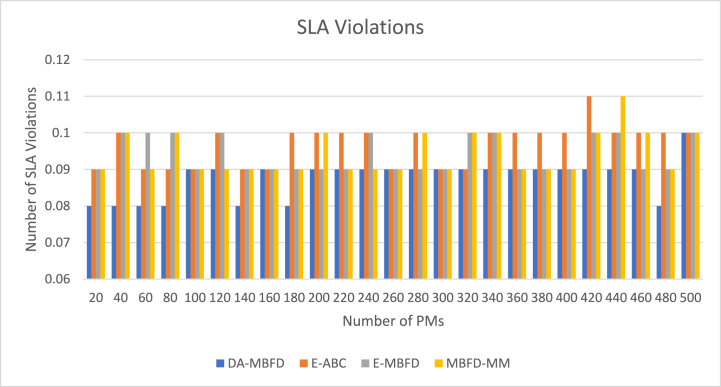
SLA violation.

The SLA violations shown by each of the implemented works are illustrated using the graph shown in Fig. 9. It is observed that with a rise in the number of PMs, a variable number of violations have been observed by each of the techniques.

**Fig. 10:**
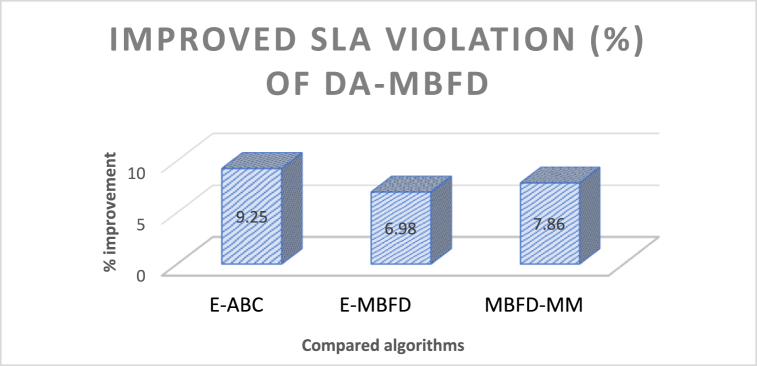
Improvement in SLA violation.

DA can maximize its performance by applying a differential evolution technique and adaptive learning parameters. Fig. 10 presents the % improvement of DA-MBFD in SLA Violation values corresponding to the number of VMs. DA-MBFD shows 9.25 %, 6.98 %, and 7.86 % minimized SLA Violation against E-ABC, E-MBFD, and MBFD-MM respectively. This reflects that with improved selection process DA-MBFD results in least SLA violations and thus shows improvement over the existing studies used in the comparative analysis.

**Fig. 11:**
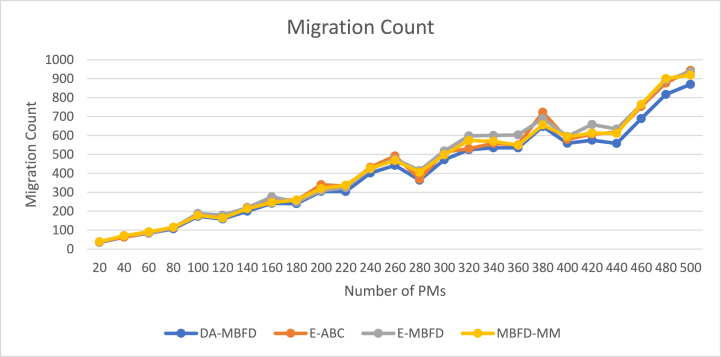
Migration count.

The variation in the migration count is given in Fig. 11. There is a clear correlation between the growth in PMs and VMs in the network and the number of VM migrations. However, DA-MBFD has an optimal number of migrations among all.

**Fig. 12:**
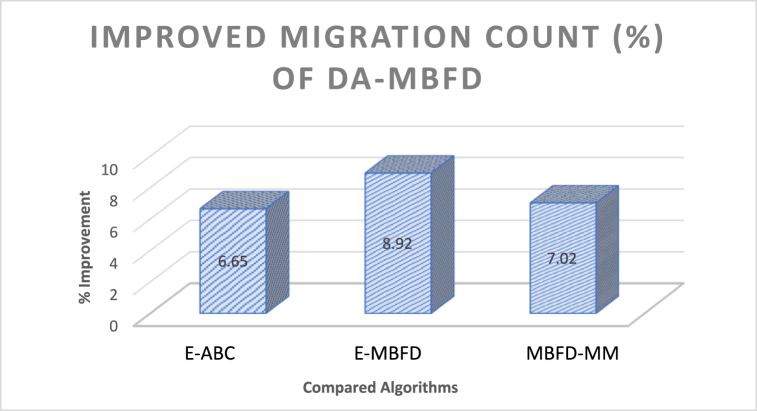
Improvement in migration count.

Fig. 12 illustrates the % improvement of migrations associated with the increasing number of VMs. An effective migration policy strives to minimize the migration count by selecting the most suitable host, while also adhering to the minimum power consumption criteria. Based on the experimental results, the % improvement in several migrations of DA-MBFD is 6.65 %, 8.92 %, and 7.02 % against E-ABC, E-MBFD, and MBFD-MM respectively. This shows using proposed DA-MBFD approach has significantly improved the process of VM allocation and migration that has significantly reduced the number of migrations and hence shown improvement over the existing approaches. The list of abbreviations and Notations is given in the end of the paper as Table 4 for better understanding of the paper.

**Table 4:** List of Notations used in the Paper.

ABBREVIATIONS AND NOTATIONS	EXPANDED FORM
CDC	Cloud Data Centers
CSPs	Cloud Service Provider
PMs	Physical Machines
VMs	Virtual Machines
MM	Minimization of Migrations
SI	Swarm Intelligence
SLA	Service Level Agreement
DA	Dragonfly Algorithm
MBFD	Modified Best Fit Decreasing
DA-MBFD	Dragonfly Algorithm with Modified Best Fit Decreasing
P_i_	Power Consumption
C_f_	Clock Frequency
U_i_	CPU Utilization
R_A_	Available Resources
PR_p_	Remaining Physical Resources of pth PM
OR	Original number of Resources
TA	Total number of Allocations to pth PM
RCR	Request Completion Ration
RC	Request Completed by PM without SLA Violation
AR	Total number of Allocated Resources
VM_R_	Number of VM request submitted
P_s_	Power Model of a Server
P_static_	Power Consumed by Static Server
P_dynami_	Power Consumed by Dynamic Server
P_total_	Total Power Consumed by a Server
A	Alignment
C	Cohesion
S	Separation
F	Attraction to Food
E	Distraction form Enemy
ΔP	Step Vector where P is the Position Vector
s	Separation of weight
a	Alignment_weight_
c	Cohesion_weight_
f	Food_Factor_
e	Enemy_Factor_
w	Inertia weight
t	Number of iterations

## 5 Conclusion and future scope

Energy efficiency has become a critical concern in modern computing systems in recent years which extends from a single server to a CDC. These CDCs consume a substantial amount of energy to support server operations for both CSPs and users. Therefore, management of the consumed energy is very important which requires better resource utilization that can be achieved by a proper VM placement strategy. This paper focuses on an effective VM placement technique termed DA-MBFD which uses the MBFD algorithm for prioritizing VMs based on their resource requirement, followed by the MM algorithm that detects the hotspot and classifies the host as normal, overutilized, and underutilized. These are then followed by a swarm-based approach (DA) that identifies overloaded hosts and migrates VMs from overloaded to underloaded hosts, maximizing energy utilization in CDCs. The proposed work shows improvement in the results when compared with E-MBFD, MBFD-MM, and E-ABC in terms of power consumption, SLA Violation, and migration count. DA-MBFD has 8.21 %, 8.6 %, 6.77 % improvement in power consumption, 9.25 %, 6.98 %, 7.86 % minimized SLA Violation, 6.65 %, 8.92 %, 7.02 % improvement in migration count compared to E-ABC, E-MBFD, MBFD-MM respectively.

The future scope of this work presents a wide range of possibilities for leveraging machine learning techniques to revolutionize resource optimization in CC environments. By integrating machine learning algorithms with historical data and advanced analytics, we can unlock valuable insights that facilitate more informed decision-making in the VM selection process. These algorithms can analyze patterns and relationships within the data to develop sophisticated models that accurately predict resource demands based on workload characteristics, historical usage patterns, and other relevant factors. Furthermore, machine learning models can be trained to dynamically adjust resource allocations in real time, considering factors such as network traffic, workload fluctuations, and performance metrics. This adaptive resource management approach ensures optimal resource utilization by effectively allocating VMs to underutilized PMs and preventing resource congestion. The future scope of this work can be characterized by the fusion of machine learning techniques with resource optimization in CC. This integration empowers CSP to leverage historical data, predictive analytics, and adaptive resource management strategies to achieve efficient resource utilization, anticipate future demands, automate decision-making processes, and balance conflicting objectives. By embracing these advancements, the CC industry can unlock unprecedented levels of scalability, performance, and cost-effectiveness, ensuring optimized resource allocation and enhancing the overall user experience.

## Data availability section

Has data associated with your study been deposited into a publicly available repository?

NO.

Has data associated with your study been deposited into a publicly available repository?

Data will be made available on request.

## CRediT authorship contribution statement

**Sindhu Rashmi:** Writing – original draft, Visualization, Validation, Software, Resources, Methodology, Investigation, Formal analysis, Data curation, Conceptualization. **Vikas Siwach:** Writing – review & editing, Visualization, Validation, Software, Resources, Methodology, Investigation, Formal analysis, Data curation, Conceptualization. **Harkesh Sehrawat:** Writing – review & editing, Visualization, Validation, Software, Resources, Methodology, Investigation, Formal analysis, Data curation, Conceptualization. **Gurbinder Singh Brar:** Writing – review & editing, Visualization, Validation, Software, Resources, Methodology, Investigation, Formal analysis, Data curation, Conceptualization. **Jimmy Singla:** Writing – review & editing, Visualization, Validation, Software, Resources, Methodology, Investigation, Formal analysis, Data curation, Conceptualization. **N.Z. Jhanjhi:** Writing – review & editing, Visualization, Validation, Supervision, Software, Resources, Project administration, Methodology, Investigation, Formal analysis, Data curation, Conceptualization. **Mehedi Masud:** Writing – review & editing, Visualization, Validation, Software, Resources, Methodology, Investigation, Formal analysis, Data curation, Conceptualization. **Mohammad Shorfuzzaman:** Writing – review & editing, Visualization, Validation, Software, Resources, Methodology, Investigation, Funding acquisition, Formal analysis, Data curation, Conceptualization.

## Declaration of competing interest

The authors declare that they have no known competing financial interests or personal relationships that could have appeared to influence the work reported in this paper.
